# The efficacy of antimicrobial therapies in the treatment of mixed biofilms formed between *Candida albicans* and *Porphyromonas gingivalis* during epithelial cell infection in the aspiration pneumonia model

**DOI:** 10.1007/s00430-025-00818-2

**Published:** 2025-02-04

**Authors:** Grazyna Bras, Ewelina Wronowska, Miriam Gonzalez-Gonzalez, Magdalena Juszczak, Magdalena Surowiec, Wiktoria Sidlo, Dorota Satala, Kamila Kulig, Justyna Karkowska-Kuleta, Joanna Budziaszek, Joanna Koziel, Maria Rapala-Kozik

**Affiliations:** 1 Department of Comparative Biochemistry and Bioanalytics, Faculty of Biochemistry, Biophysics and Biotechnology, Jagiellonian University, Gronostajowa 7, Kraków, 30-387 Poland; 2 Doctoral School of Exact and Natural Sciences, Faculty of Biochemistry, Biophysics and Biotechnology, Jagiellonian University, Gronostajowa 7, Kraków, 30-387 Poland; 3 Department of Microbiology, Faculty of Biochemistry, Biophysics and Biotechnology, Jagiellonian University, Gronostajowa 7, Kraków, 30-387 Poland

**Keywords:** In vitro biofilm, Aspiration pneumonia, Antibiotic and antimycotic treatment, *Candida albicans*, *Porphyromonas gingivalis*, Host cell responses

## Abstract

Aspiration pneumonia is a serious respiratory condition, which is particularly prevalent in patients with dysphagia, neurological disorders, or those undergoing surgical interventions. The formation of multispecies biofilms in the oral cavity, involving the bacterial periodontopathogen *Porphyromonas gingivalis* and the opportunistic pathogenic fungus *Candida albicans*, may also be related to the development of this serious disease, contributing also to the resistance to standard antimicrobial treatment. Therefore, this research aimed to evaluate the efficacy of selected antibiotics‒levofloxacin, metronidazole, meropenem, vancomycin‒and antifungal agents‒amphotericin B, caspofungin, and fluconazole‒on these mixed biofilms in the aspiration pneumonia model. While metronidazole and levofloxacin effectively inhibited bacterial viability in the mixed biofilms, lower doses increased release of bacterial proteases. In the conditions of mixed biofilms meropenem and vancomycin showed reduced efficacy, requiring significantly higher doses to achieve similar effect in mixed biofilms as in single bacterial cultures. Treatment with antifungals revealed that amphotericin B significantly impacted fungal cell viability within mixed biofilms, and this effect was enhanced when the antifungal drug was applied in the presence of *P. gingivalis*. Caspofungin and fluconazole showed variable efficacy, with caspofungin being more effective against *C. albicans* cells within biofilm.

These findings indicated that due to the mutual microbial protection in the mixed-species biofilm, *P. gingivalis* retained its virulence despite increasing antibiotic doses. However, no excessive benefit of mixed biofilms was observed for *C. albicans* in the presence of antifungals, indicating the minor importance of yeasts in aspiration pneumonia development and their protective role for other pathogens in mixed-species infection.

**Graphical abstract:**

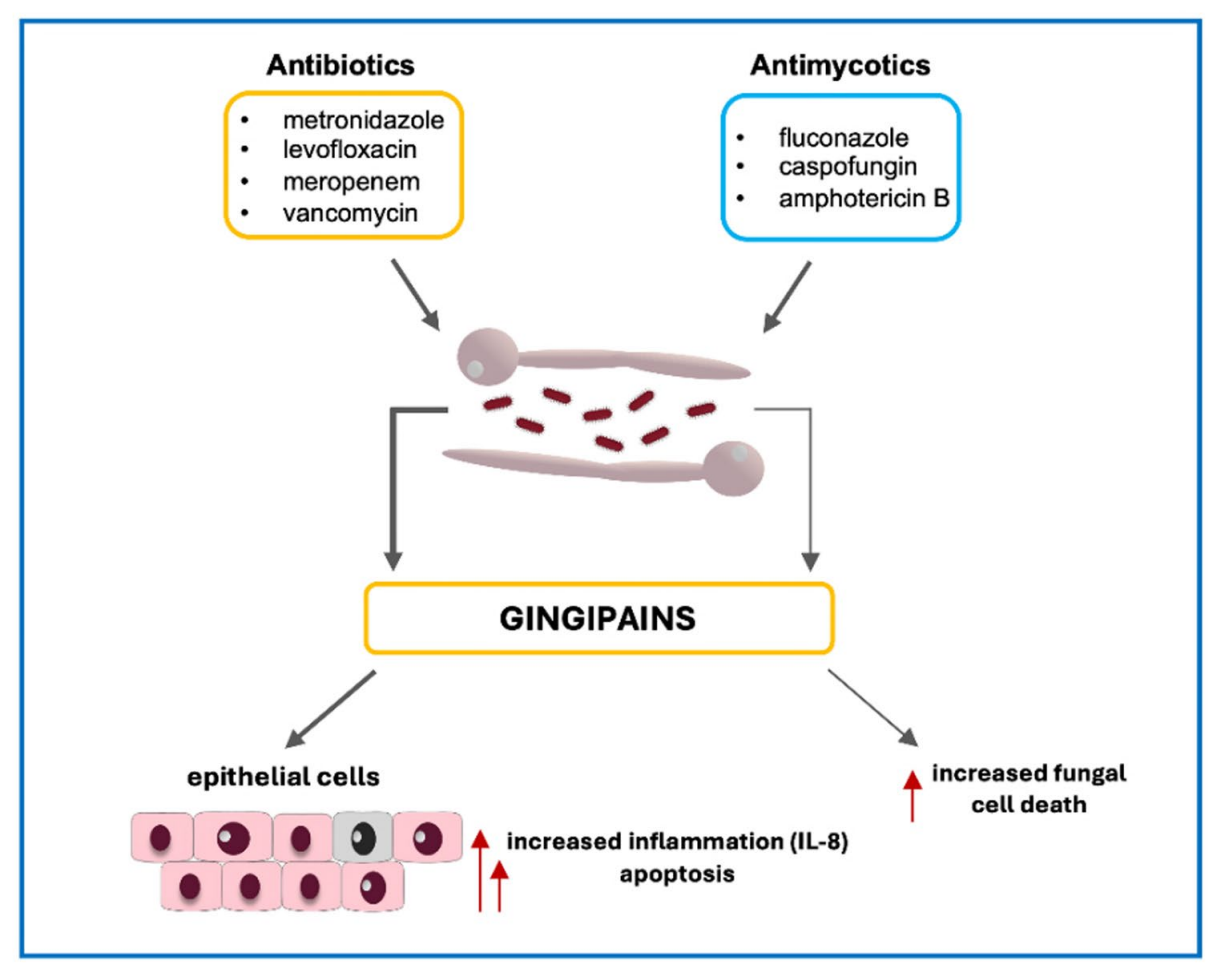

**Supplementary Information:**

The online version contains supplementary material available at 10.1007/s00430-025-00818-2.

## Introduction

The formation of complex multispecies biofilms by a range of microorganisms is often cited as a major cause of infection resistance to treatment with available antimicrobial therapies. This resistance results from the heterogeneous, complex, and specific interactions that occur in the biofilm and are difficult to control. They relate to various types of relationships exhibited by coexisting species, including symbiotic, mutualistic, synergistic, or antagonistic interactions [1].

The human oral cavity is the key site for the formation of mixed-species biofilm. This complex microecosystem encompasses a great diversity of microorganisms, including bacteria, fungi, viruses, mycoplasma, and protozoa. Maintaining a proper balance of the oral microbiota is critical for promoting not only oral health but also overall systemic health. It has been previously suggested, that dysbiosis in the oral microbiota might contribute to the development of various diseases, including oral conditions like dental caries and periodontitis, as well as systemic diseases such as diabetes [2], bacteremia [3], endocarditis [4], rheumatoid arthritis [5], atherosclerosis [6], or Alzheimer’s disease [7]. Many studies also documented an association between oral health and the risk of respiratory diseases such as pneumonia, chronic obstructive pulmonary disease, and lung cancer [8–10], where microbes can be aspirated into the lower respiratory tract, infecting the lung and trachea, and finally causing diseases, especially under conditions of low host resistance [11, 12].

One of the less frequently considered issues in this regard is the risk of the development of aspiration pneumonia (AP), that affects patients with dysphagia resulting from the elderly, neurological disorders, surgical interventions and anesthesia or endotracheal intubation [13]. Importantly, not only does the oral microbiome serve as a reservoir of opportunistic respiratory pathogens, but also pathogens existing in the local environment of the oral microecosystem and found in dental plaque and periodontal pockets, especially in patients with poor dental hygiene. This can lead to the selection of other types of AP characterized as ventilator-associated pneumonia, hospital-acquired pneumonia, or community-acquired pneumonia [14, 15].

Periodontal diseases have been recognized as important risk factors for AP, with the participation of the major pathogen that comprises the red complex bacteria *Porphyromonas gingivalis* [16]. *P. gingivalis* pathogenicity is reflected in an arsenal of virulence factors (proteinases, fimbriae, lipopolysaccharide) responsible for host defense system inactivation, as well as in the moderation of the environmental microbial community toward successful host cell colonization and/or destruction [17]. *P. gingivalis* can form a mixed-species biofilm with different types of bacteria and also with fungi, especially *Candida albicans*, which was also detected in the typical location of periodontal pathogens [18, 19]. Their mutual coexistence is based on the interactions of cell surface adhesins, involving fungal adhesin Als3 and bacterial protease RgpA [20] or bacterial internalin-family protein InlJ [21], and include the modification of *C. albicans* cell surface proteins by bacterial peptidylarginine deiminase (PPAD) [22]. These interactions might help *P. gingivalis* find the most favorable growing conditions and protection against host recognition at the initial stage of infection [23, 24].

Nevertheless, the role of *C. albicans* in AP is still unclear. Thus far, it has been assumed that *C. albicans* yeasts are very rarely involved in AP, and their detection in samples has been considered a contaminant arising from imprecise material collection from the infection site. However, since microbial identification methods have become more sensitive and sample collection methods more precise, current studies are paying more attention to the possible pathogenic and contributory role in the etiology of pneumonia for *Candida* species in combination with bacterial pathogens, especially in patients with a history of chronic aspiration [25–28]. Furthermore, it is critical to validate the efficiency of typical antibiotic and antimycotic therapies commonly applied in AP in light of dual-species biofilm formation with mutual protective properties of the involved species, resulting in the treatment complications or failures. Therefore, the main objective of this study was to verify the effectiveness of selected antibiotics and antimycotics recommended in the treatment of AP, concerning infections involving mixed biofilms formed by *P. gingivalis* and *C. albicans*, that can be present in immunosuppressed patients with coexisting periodontal diseases.

## Materials and methods

### Bacterial and yeast strains and their growth conditions

*P. gingivalis* wild-type strain W83 (ATCC BAA-308) purchased from American Type Culture Collection (ATCC; Manassas, VA, USA) and an isogenic gingipain-null mutant, *∆K∆RAB* (lacking gingipains Kgp, RgpA, and RgpB), obtained as described previously [16], were cultured under anaerobic conditions (90% N₂, 5% CO₂, 5% H₂) at 37 °C on blood (5% (v/v) sheep blood) agar plates (BTL, Lodz, Poland) or in liquid tryptic soy broth (TSB) (30 g/l; Sigma-Aldrich, St Louis, MO, USA) with yeast extract (5 g/l; Bioshop, Burlington, Canada) supplemented with hemin (5 µg/ml), L-cysteine (50 µg/ml), and vitamin K (0.5 µg/ml) (all from Sigma-Aldrich), and additionally with tetracycline (1 µg/ml; Sigma-Aldrich) in the case of the *∆K∆RAB* mutant. Bacteria from overnight liquid culture were centrifuged (4500 × *g*, 30 min) at 4 °C, washed three times, and resuspended in phosphate buffered saline, pH 7.4 (PBS; Biowest, Nuaillé, France). The optical density (OD) at 660 nm was measured with the use of a Shimadzu UVmini-1240 spectrophotometer (Shimadzu, Kyoto, Japan) to estimate the number of bacterial cells.

*C. albicans* strain 3147 (ATCC 10231) was purchased from American Type Culture Collection. Yeast cells were grown on YPD (1% yeast extract, 2% soybean peptone, and 2% glucose) agar (1.5%) plates (all components from Sigma-Aldrich) for 24 h at 30 °C and in liquid YPD medium for 18 h at 30 °C with shaking (170 rpm) using an orbital rotary shaker MaxQ 6000 (Thermo Fisher Scientific, Waltham, MA, USA) in aerobic conditions. Fungal cells from liquid cultures were harvested by centrifugation (3000 × *g*, 3 min), washed three times with PBS, and counted using OD measurements at 600 nm.

### Single and mixed species biofilm formation

Biofilms were formed on the surface of flat-bottomed 96-well microplates (Corning, Glendale, AZ, USA). *P. gingivalis* and *C. albicans* cells were washed with PBS and suspended in RPMI 1640 medium buffered with 25 mM HEPES, pH 7.3 (Biowest), supplemented with 10% heat-inactivated fetal bovine serum (FBS; Gibco, Grand Island, NY, USA). Two independent suspensions were prepared: 2 × 10⁸ *P. gingivalis* cells /ml and 2 × 10⁷ *C. albicans* cells/ml. The wells of the plate were inoculated with 100 µl of each suspension for mixed biofilm formation or with 100 µl of one of the suspensions and the same volume of appropriate sterile medium (described above) to prepare single-species biofilms. The biofilms were incubated under aerobic conditions in the New Brunswick Galaxy 170R CO₂ incubator (Eppendorf, Hamburg, Germany) without shaking at 37 °C for 24 h.

For the purpose of standardizing the conditions for biofilm formation (number of microbial cells seeded in the wells, presence of FBS in the medium, and time of biofilm formation) with optimal protection of bacteria against aerobic conditions, evidenced by the highest gingipain production, a combination of 10^6^ or 10^7^* C. albicans* cells/ml and 10⁸ *P. gingivalis* cells/ml, or each microorganism separately, were placed in RPMI 1640 with or without 10% FBS in the wells of a 96-well microplate. Then, biofilms were formed for 24 and 48 h at 37 °C under aerobic conditions.

### Treatment of biofilms with antibiotics and antimycotics

Antibiotics and antifungal agents were added to the 24-hour-old mixed and single biofilms formed under aerobic conditions in a 5 µl volume to achieve final concentrations of levofloxacin (Sigma-Aldrich, 10 mg/ml stock in DMSO) at 0.1, 1, 10, or 50 µg/ml; meropenem (Sigma-Aldrich, 5 mg/ml stock in H₂O) at 0.1, 1, 10, or 50 µg/ml; metronidazole (Sigma-Aldrich, 10 mg/ml stock in H₂O) at 0.1, 1, 5, or 10 µg/ml; vancomycin (Sigma-Aldrich, 100 mg/ml stock in DMSO) at 0.1, 1, 5, 10, or 50 µg/ml; amphotericin B (Biowest, 250 µg/ml stock in 35% sodium deoxycholate and 10% phosphate buffer) at 0.25, 0.5, 1, or 2 µg/ml; caspofungin (Sigma-Aldrich, 10 mg/ml stock in H₂O) at 0.01, 0.1, 0.5, or 5 µg/ml; and fluconazole (Sigma-Aldrich, 20 mg/ml stock in DMSO) at 0.5, 5, or 50 µg/ml. Incubation of the biofilms with added drugs or biofilms without drugs (controls) was performed for 24 h under the same conditions as before treatment. The tests were performed at least three times in duplicate.

In another type of experiment, the combinations of antibiotic and antimycotic (levofloxacin with amphotericin B) were also added to the 24-hour-old biofilms formed under aerobic conditions. Both the administration of drugs and the further incubation of the biofilms were carried out as in the case of treatment of the biofilms with a single drug.

### Gingipain activity measurement

Supernatants collected from 24-hour-old biofilms or from 48-hour-old biofilms untreated and treated with drugs were separated from the microbial cells by multiple centrifugation steps (once at 3000 × *g* for 5 min and three times at 6000 × *g* for 15 min). The proteolytic activity of gingipain R (Rgp) in each of the supernatants was determined by monitoring the hydrolysis of the chromogenic substrate benzoyl-l-arginine-p-nitroanilide (BApNA; Sigma-Aldrich), as previously described [29] with minor modifications. Briefly, 70 µl of assay buffer (200 mM Tris-HCl, 100 mM NaCl, 5 mM CaCl₂, pH 7.6) supplemented with fresh 10 mM L-cysteine was mixed with 30 µl of supernatant in the wells of a microplate (Sarstedt, Nümbrecht, Germany). After 5-minute preincubation at 37 °C, 20 µl of BApNA (final concentration 0.5 or 4 mM) was added to the wells. The formation of p-nitroaniline was measured as an increase in the absorbance at 405 nm over a period of 1–2 h (with absorbance readings taken every minute) using a Synergy H1 microplate reader (BioTek Instruments, Winooski, VT, USA).

### Metabolic activity measurement

To determine the metabolic activity of microbial cells in biofilms, the 2,3-bis(2-methoxy-4-nitro-5-sulfo-phenyl)-2 H-tetrazolium-5-carboxanilide (XTT; Invitrogen, Waltham, MA, USA) reduction assay was used. The tests were performed as described previously [30] with some modifications. 24- or 48-hour-old single and mixed biofilms were washed once with PBS and 100 µl of RPMI 1640 without phenol red (Biowest) and 50 µl of a fresh mixture of XTT at a final concentration of 1 mg/ml and phenazine methosulfate (PMS; Sigma-Aldrich) at a final concentration of 5 µg/ml were added. After 40 min of incubation at 37 °C in the dark under aerobic conditions for mixed and monospecies *C. albicans* biofilms, 100 µl of supernatants were transferred to the wells of a new microplate (Sarstedt). The absorbance of the formazan product was measured at 450 nm using a Synergy H1 microplate reader (BioTek Instruments).

### Microbial viability determination

To determine the viability of biofilm-forming microbial cells, a colony-forming unit (CFU) assay was used. The 48-hour-old biofilms were washed once, resuspended in PBS, scraped from the wells, homogenized by vortexing, and diluted ten thousand times in PBS. Then, 25 µl of the final suspensions were plated in quadruplicate on appropriate solid medium - YPD agar to determine the number of *C. albicans* cells, and blood agar to determine the number of *P. gingivalis* cells. They were cultivated aerobically at 30 °C for 24 h and anaerobically (in a GENbox jar anaerobic generator (bioMérieux, Craponne, France) at 37 °C for 5 days, respectively. After incubation, the numbers of colonies were counted. Each experiment was carried out at least three times in duplicate.

### Permeabilization assay

After treatment with amphotericin B, the 48-hour-old mono- and dual-species biofilms were washed once with PBS, suspended in PBS containing 1 µM Sytox™ Orange Nucleic Acid Stain (Thermo Fisher Scientific, Waltham, MA), and incubated for 5 min at room temperature in the dark. The permeabilization of the fungal membrane was visualized using an Olympus IX73 microscope (Olympus, Tokyo, Japan) equipped with a Hamamatsu Orca Spark camera (Hamamatsu, Hamamatsu City, Japan) and a UPLXAPO60XO lens (Olympus). All images were taken using the same parameters (exposure time, spectrum range), enabling the comparison of the fluorescence of the dye between different preparations, prepared in the form of Z-stacks (50 μm) and subjected to 3D deconvolution using CellSense software (Olympus).

### Detection of metacaspase activation

The metacaspase activity was determined using a method previously described by Guevara-Lora et al. (2023) [31]. The 48-hour-old biofilms treated with amphotericin B were washed with PBS, suspended in 100 µl of staining solution containing CaspACE™ FITC-VAD-FMK In Situ Marker (Promega, Madison, WI, USA) at a concentration of 10 µM, and incubated for 1 h at room temperature in the dark under aerobic conditions. After this time, the biofilms were washed four times and then suspended in PBS. The green fluorescence of the marker bound to the active metacaspases was visualized using an Olympus IX73 microscope as described above.

### Host cell culture and stimulation

Human bronchial epithelial BEAS-2B cells (ATCC CRL-9609) were purchased from ATCC. The cells were cultured in serum- and gentamycin-free BEGM medium (Lonza, Levallois-Perret, France) at 37 °C in an atmosphere of 5% CO_2_ and 95% humidity in bottles coated with 0.05 mg/ml of collagen (Sigma-Aldrich). Before each experiment, cells (4 × 10^4^) were seeded in the collagen-covered wells of 96-well glass-like microplate (Cellvis, Sunnyvale, CA, USA) and cultured in BEGM for 24 h to 90% confluence. The medium was removed and 100 µl of the mixture of RPMI 1640 with 20% supernatant collected from 48-hour-old mixed biofilms (untreated or treated with levofloxacin (0.1–10 µg/ml) or levofloxacin and amphotericin B (0.5 µg), previously filtered with Ultrafree-CL Centrifugal Filters with a pore size of 0.22 μm (Merck, Darmstadt, Germany), was added to the cell monolayers. Then, BEAS-2B cells were stimulated for 24 h. Cells incubated in RPMI 1640 with 2% FBS, and in the same medium supplemented with levofloxacin (10 µg/ml) or combination of levofloxacin (10 µg/ml) and amphotericin B (0.5 µg/ml), served as controls.

### Apoptosis and host cell death analysis

After a 24-hour incubation with sterile biofilm supernatants, BEAS-2B cells were washed once with PBS and then suspended in 50 mM HEPES buffer with 700 mM NaCl and 12.5 mM CaCl_2_ containing Annexin V Alexa Fluor^®^ 488 (Invitrogen, Waltham, MA) diluted twenty-fold. Cells were stained at room temperature for 15 min, then washed three times with PBS and incubated for 10 min at room temperature in RPMI 1640 medium without phenol red (Biowest) with added 1 µM Sytox™ Orange Nucleic Acid Stain. Cell visualization was performed using an Olympus IX73 microscope and the obtained images were processed with Olympus CellSens Dimension 3.1 imaging software.

### IL-8 detection assay

To measure IL-8 production levels, BEAS-2B cells were stimulated as described above, except for the concentration of biofilm-derived sterile supernatants, which was 2.5%. After 24-hour incubation, supernatants collected from above BEAS-2B cells were centrifuged to remove accidentally detached cells (once for 5 min at 3000 rpm; twice for 10 min at 7000 rpm, each time with the sediment discarded). The level of IL-8 produced by human cells was determined using the Human IL-8 ELISA Set (BD Biosciences, San Diego, CA, USA OptEIA™) strictly according to the manufacturer’s instructions.

### Statistical analysis

Statistical analyses were performed with the GraphPad Prism 8 software (GraphPad Software, San Diego, CA, United States). To evaluate the significance between groups, one-way ANOVA with Dunnett’s multiple comparison *post hoc* test was used. The results were considered statistically significant at value of *p* < 0.05: *p < 0.05, **p < 0.01, ***p < 0.001 or ****p < 0.0001.

## Results

### *P. gingivalis* protease activity as bacterial viability sensors in a mixed biofilm

**Fig. 1:**
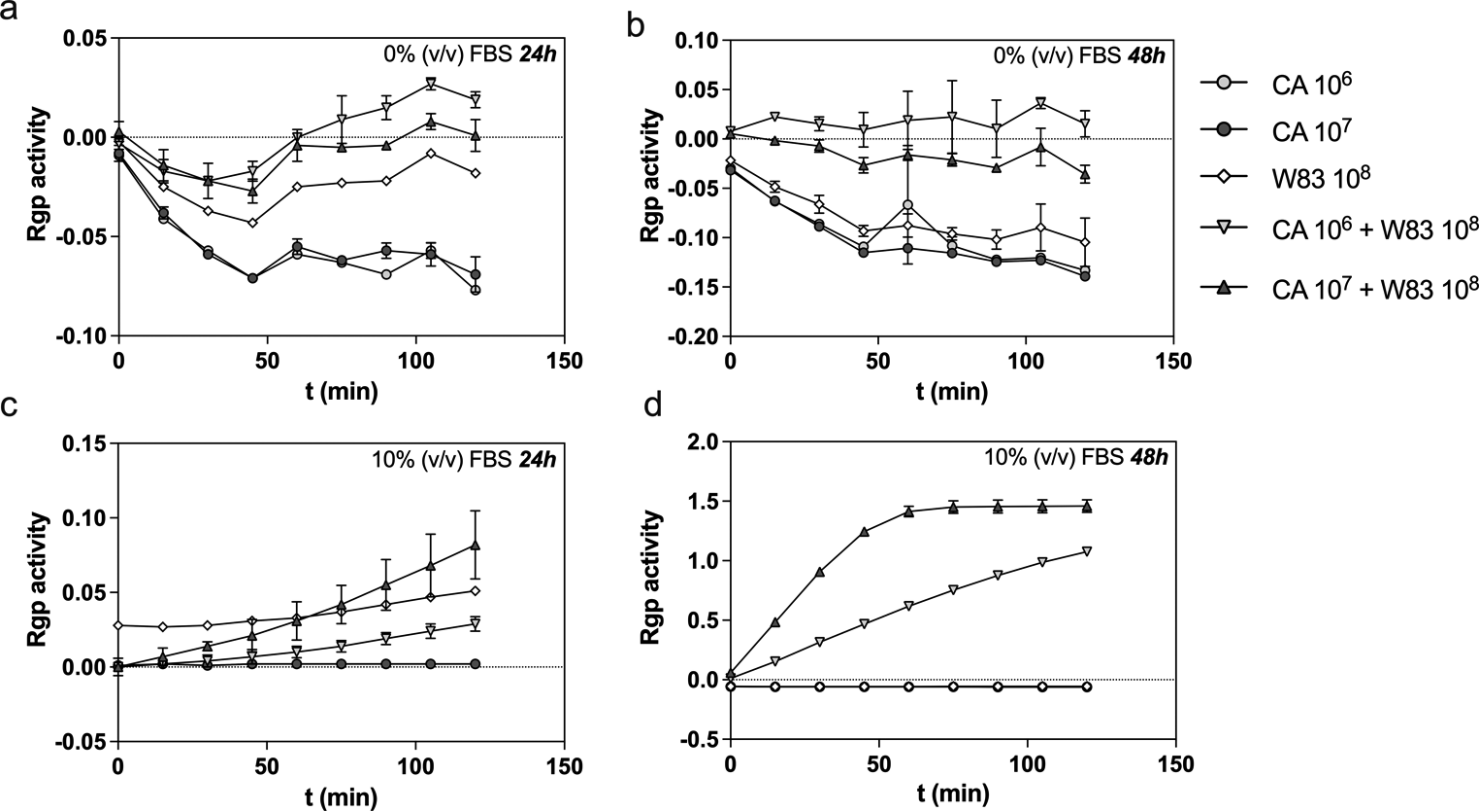
Gingipain R (Rgp) activity in supernatants collected from 24- and 48-hour-old biofilms formed under aerobic conditions by *P. gingivalis* strain W83 (W83) and *C. albicans* strain 3147 (CA) cells. The selected numbers of cells were cultured in RPMI 1640 medium to form biofilm (10^6^ or 10^7^ CA and/or W83 10^8^ cells per ml) in the absence (0%) (graphs a, c) or in the presence (10%) of FBS (graphs b, d). The activity of Rgp was measured with the BApNA substrate. The data shown (after subtracting the results for the reference sample) are presented as mean ± SEM post hoc test

The model we implemented for considering the effects of antibacterial or antifungal drugs on a mixed biofilm assumed a scenario in which both microorganisms are in initial contact under aerobic conditions for 24 h. As previously indicated, during the development of mixed biofilm with *C. albicans*, a hypoxic microenvironment was created to support the growth of anaerobic bacteria [32]. The efficient functioning of the *P. gingivalis* cells under these conditions and their further growth can be characterized by increased bacterial proteolytic activity, resulting from the production of gingipains. This parameter – protease production – was used in further verification of the bacterial viability within the mixed-species biofilm developed over an additional 24 h, with or without antimicrobial treatment. As a host tissue-associated opportunistic pathogen, *C. albicans* often encounters albumin during commensalism or other serum proteins when causing infections accompanied by increased vascular permeability. To mimic different stages of mucosal infection, we introduced FBS (10%) in our model. FBS can influence hyphae formation and the ability of *C. albicans* to damage host cells [29]. Moreover, FBS was also critical to maintain the viability of bacterial cells under aerobic conditions and correlated with increasing gingipain production by *P. gingivalis* (Fig. 1).

As demonstrated in Fig. 1, the gingipain activity was very low after 24 h of biofilm formation and increased significantly during the next 24 h of microbial co-existence. As we wanted to verify the efficiency of the treatment against developed biofilm, we used 24-hour growing mixed-species biofilm for its further antibacterial and antifungal treatment and analyzed the effectiveness of therapy after the next 24 h of intervention. This approach allowed us to control the efficacy of the therapy at the level of production of one of the main bacterial virulence factors. Moreover, it reflects the use of drugs and treatment on an already pre-formed biofilm, and not at the stage of its initiation, which is crucial due to the actual clinical value of the obtained observations, as the treatment is often introduced at the stage when the infection is already observable and diagnosable.

### Effectiveness of antibacterial drugs on bacterial functionality in mixed biofilms

Since the antibiotic efficiency parameters, MIC50 and MIC90, depend on the type of *P. gingivalis* strain used [33, 34], we performed standardization of the action of selected antibiotics‒metronidazole, levofloxacin, meropenem, vancomycin‒on *P. gingivalis* strain W83 used herein by culturing the bacteria in RPMI 1640 medium and under anaerobic conditions (Fig. 1 in the Supplementary data). These determinations were important to compare their variability within the mixed-species biofilm formed in the presence of 10% FBS, which can influence the availability of applied compounds (Table 1 in the Supplementary data).

The analysis of antibiotic efficacy against mixed-species biofilms was performed with regard to *P. gingivalis* cells, measuring their viability using the CFU assay, and metabolic functionality represented by gingipain activity measured in the culture supernatant. The entire biofilm, involving both types of cells, was also analysed for global metabolic activity using the XTT test. As bacterial proteolytic activity can influence fungal cell condition, the viability of fungal cells was also assessed using the CFU assay.

**Fig. 2:**
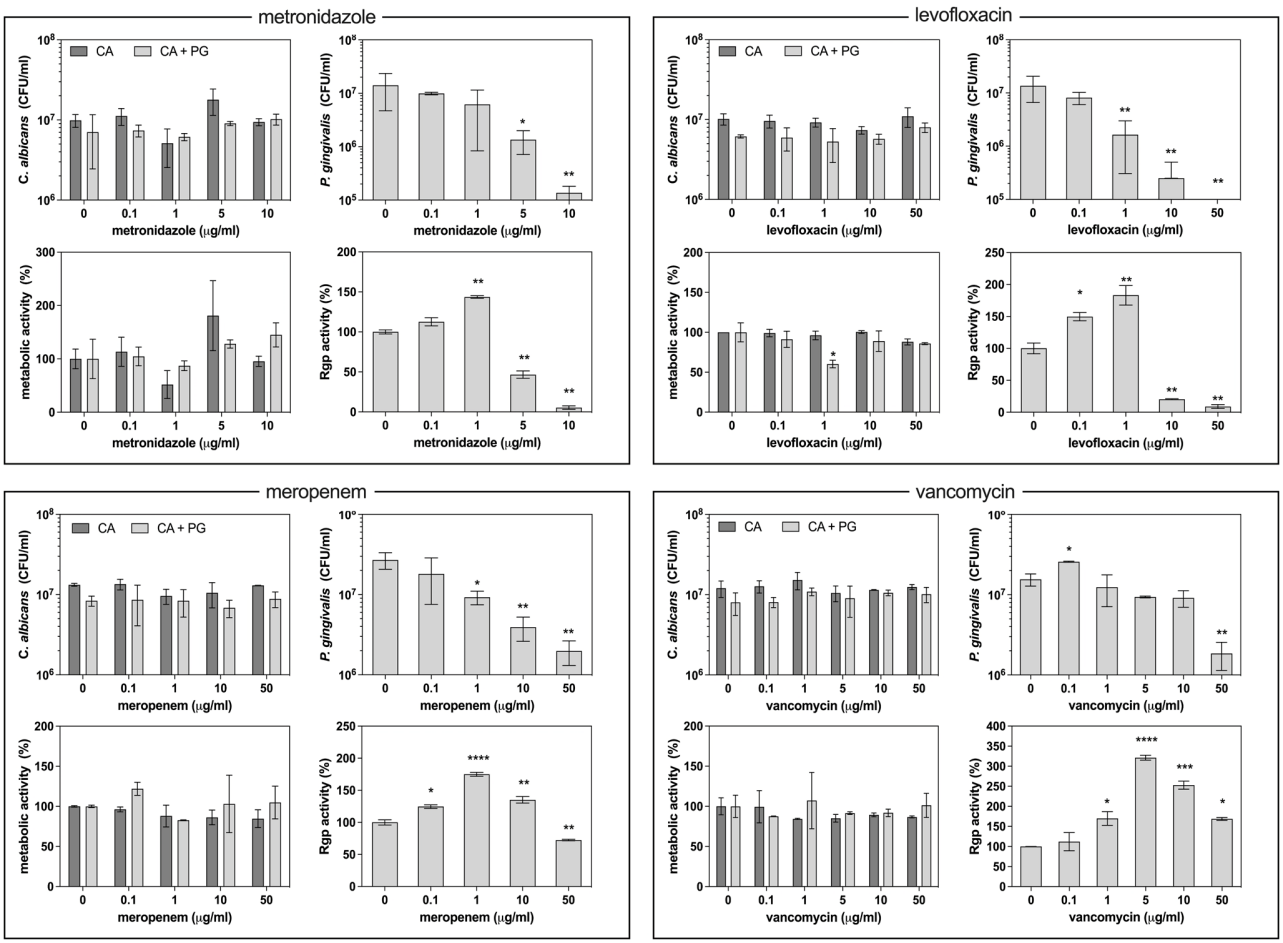
Effect of antibiotic treatment on biofilm formation by *C. albicans* and *P. gingivalis* under aerobic conditions. *C. albicans* (10^7^ cells/ml) and *P. gingivalis* (10^8^ cells/ml) were cultured in RPMI 1640 medium with 10% FBS for 24 h at 37 °C under aerobic conditions, then treated with antibiotic and incubated for the next 24 h. In the washed biofilms, CFU/ml of *C. albicans* and *P. gingivalis* was determined by CFU assay and the metabolic activity of cells by the XTT reduction assay. In the supernatants, the gingipain R (Rgp) activity was measured. Representative results from five independent experiments are presented as mean ± standard error. Statistical analysis was performed using a one-way ANOVA with Dunnett’s post hoc test to compare the means of groups (* *p* < 0.05; ** *p* < 0.01; *** *p* < 0.001; **** *p* < 0.0001)

Among the antibiotics analysed, the most efficient against bacteria in the mixed biofilm were metronidazole and levofloxacin (Fig. 2). Mixed biofilm treatment with metronidazole resulted in a MIC90 value of 5 µg/ml, and this did not differ significantly compared to the effectiveness of metronidazole against monospecies biofilm. At this concentration, gingipain production was completely inhibited. However, the gradual inactivation of *P. gingivalis* viability did not correspond with a similar change in Rgp activity. At low concentrations of the metronidazole, where the bacteria still retained their viability, there was an increase observed in the gingipain activity by up to 50% compared to bacteria not treated with any agent. At the same time, *C. albicans* cell viability was not significantly influenced. Analysis of the metabolic activity of the biofilm showed some increase after elimination of gingipains, but the changes were not statistically significant.

Similar tendency was also observed for levofloxacin application. However, the observed MIC90 value of 1 µg/ml was about ten times higher compared to the monospecies culture-killing assay. Again, changes in bacterial viability did not correlate with the gingipain activity detected in the supernatant, as at low antibiotic concentrations, below 1 µg/ml, there was an increased release of gingipains into the medium, with their activity increasing twofold at this threshold concentration. These effects are accompanied by a decrease in the metabolic activity of the biofilm, but these changes do not translate into statistically significant differences in yeast viability, as measured by CFU values. However, biofilm metabolic activity increased after the inhibition of gingipain activity, accompanied by a slight increase in fungal cell survival.

Treatment of mixed-species biofilm with meropenem showed an analogous pattern of influence on bacterial cell viability, with an MIC90 value of 10 µg/ml, which was ten times higher compared to the treatment of only bacterial culture.

Treatment of biofilm with vancomycin presented similar trends of influence as observed for meropenem application. However, in this case, MIC90 was achieved at a concentration of 50 µg/ml, which is fifty times higher compared to the monospecies culture of *P. gingivalis*. We also observed a several-fold increase in gingipain activity in the medium, without any considerable influence on the viability of *C. albicans* cells or mixed biofilm metabolic activity.

### Impact of antifungal drugs on *C. albicans* in mixed biofilms

**Fig. 3:**
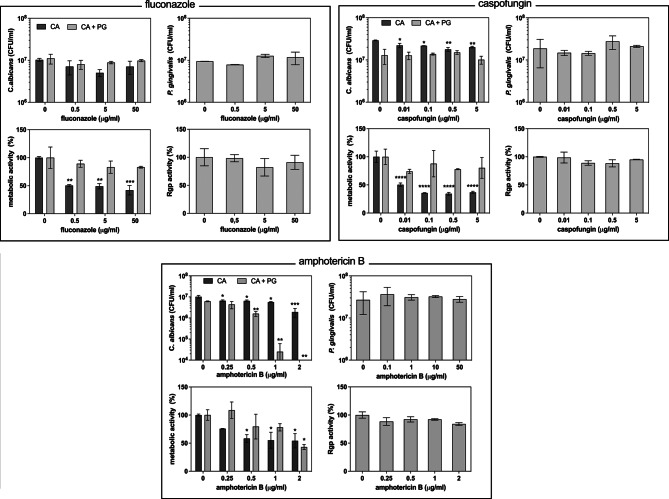
The impact of selected antifungal agents on the properties of fungal and bacterial cells within mixed biofilm formed under aerobic conditions. After 24 h of *C. albicans* (10^7^ cells/ml) and *P. gingivalis* (10^8^ cells/ml) culture in RPMI 1640 with 10% FBS at 37 °C under aerobic conditions, antifungals were added, and microbes were incubated for the next 24 h. In the washed 48 h-old biofilms, CFU/ml of *C. albicans* and *P. gingivalis* were determined, and the metabolic activity of cells was evaluated using the XTT assay. The gingipain R (Rgp) activity was measured in the supernatants. Representative results from four independent experiments are presented as mean ± standard error. Statistical analysis was performed using a one-way ANOVA with Dunnett’s post hoc test to compare the means of groups (*p < 0.05; ** *p* < 0.01, *** *p* < 0.001; **** *p* < 0.0001)

The antifungal agents selected in this study included commonly used fluconazole, caspofungin, and amphotericin B. Analysis of the condition of *P. gingivalis* cells during treatment of the mixed biofilm with fluconazole (Fig. 3) did not identify any influence on bacterial viability and gingipain production, and we also did not identify any influence of fluconazole on the viability of the mixed-species biofilm forming fungal cells, in contrast to the monospecies biofilm where a partial reduction in fungal cell viability was observed in the applied drug concentration range. The metabolic activity of the mixed biofilm was significantly increased in comparison to that of the fungal monospecies biofilm.

Although we did not find any influence of caspofungin on the condition of bacterial cells and gingipain production, the viability of fungal cells was more sensitive to the high activity of expressed gingipains. The metabolic activity of the mixed biofilm again exceeded that of the fungal biofilm under treatment.

**Fig. 4:**
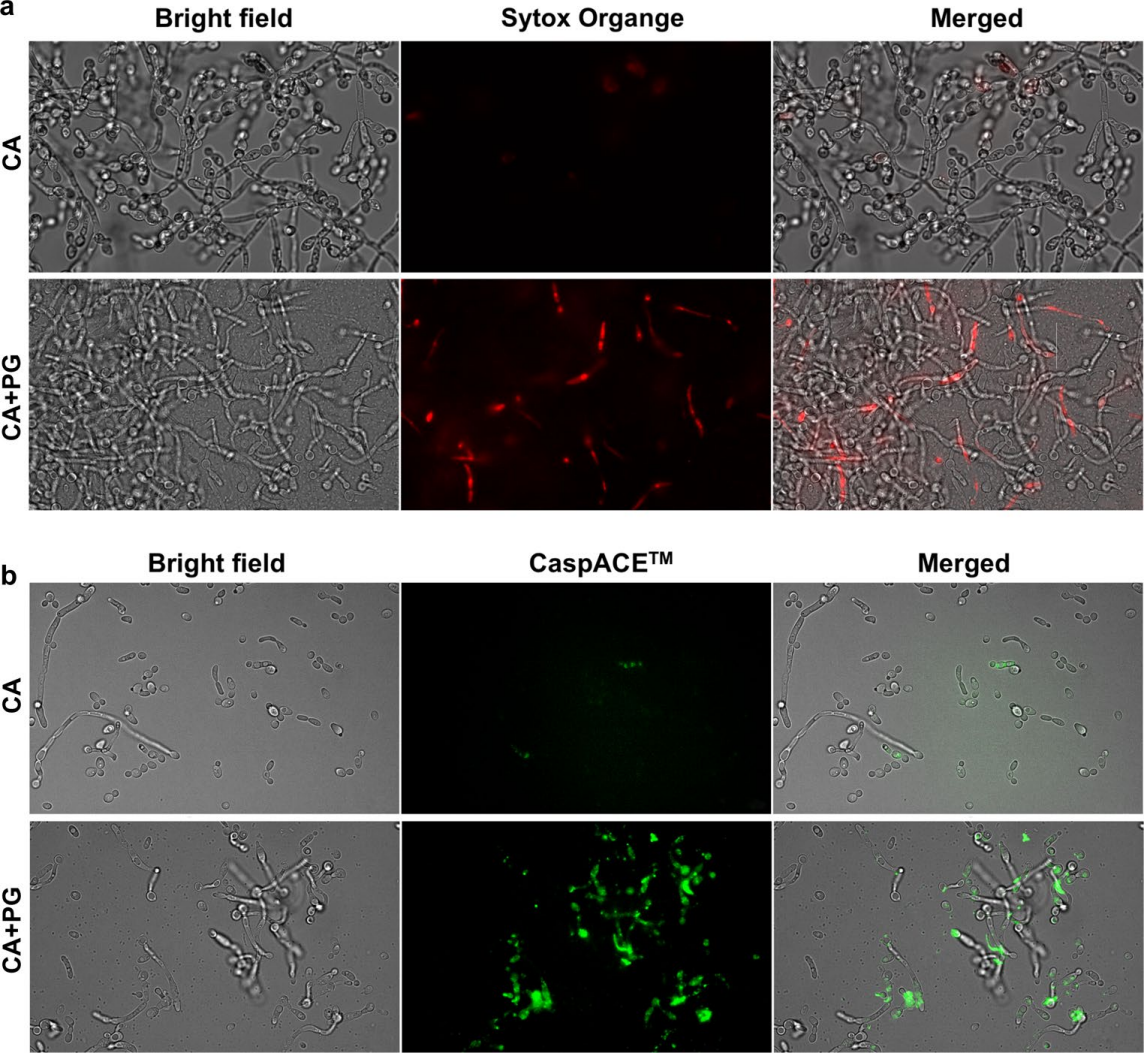
Increased permeabilization of *C. albicans* cell membrane (**a**) and metacaspase activation (**b**) under the influence of amphotericin B in mixed *C. albicans* and *P. gingivalis* biofilms. Single-species (10^7^ cells/ml of *C. albicans*) and mixed-species (10^7^ cells/ml of *C. albicans* and 10^8^ cells/ml of *P. gingivalis*) biofilms were formed in RPMI 1640 with 10% FBS at 37 °C under aerobic conditions. After 24 h, amphotericin B (2 µg/ml) was added, and incubation was continued for the next 24 h. The biofilms were stained with Sytox Orange (1 µM) to detect cell permeability. The CaspACE ™ FITC-VAD-FMK In Situ Marker kit (10 µM) was used to assess metacaspase activation. Representative images from microscopic analysis are presented

The influence of amphotericin B was observed not only for the monospecies fungal culture (MIC90 = 2 µg/ml) but even more significant for *C. albicans* cells co-cultured with *P. gingivalis* (Fig. 3 ). In this case, the fungal cells were more sensitive to the antifungal properties of amphotericin B and were killed at its lower concentration. The mixed biofilm metabolic activity decreased in similar way as observed for fungal monoculture. No changes in viability or gingipain activity were observed for bacterial cells. To verify the increased effectiveness of the antifungal properties of amphotericin B in the treatment of mixed biofilms, we compared its action on fungal cell permeability in monospecies and mixed-species biofilms, using Sytox Orange (Fig. 4A). The results were further supported by studying fungal caspase activity under these conditions (Fig. 4B). We found that 24-hour contact of fungal cells with bacteria and amphotericin B led to increased permeability of fungal cells in both yeast and hyphal morphologies, accompanied by the activation of fungal cell apoptosis.

### Consequences of applied mixed anti-biofilm therapies for host cells

**Fig. 5:**
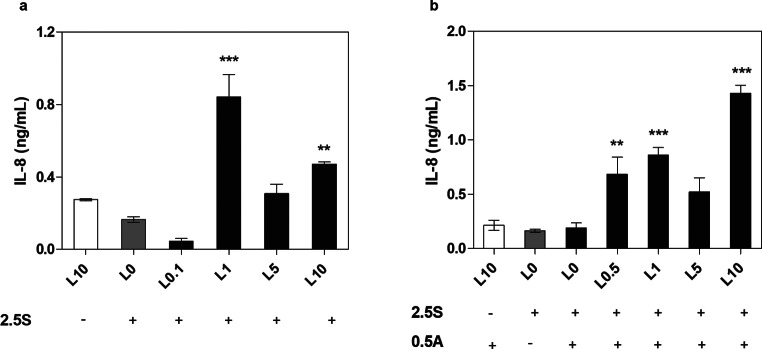
IL-8 released by BEAS-2B cells treated with supernatant produced by mixed-species biofilm of *C. albicans* and *P. gingivalis* during antibiotic and antimycotic therapy. IL-8 levels were determined in the medium collected from BEAS-2B cells incubated for 24 h with 2.5% supernatants (2.5 S) obtained from the biofilms treated with (**a**) levofloxacin (L: 0.1–10 µg/ml) or (**b**) a mixture of levofloxacin (L: 0.5–10 µg/ml) and amphotericin B (A: 0.5 µg/ml). The data shown are presented as mean ± SEM. Statistical analysis was performed with one-way ANOVA test with Dunnett’s multiple comparisons using GraphPad Prism software. The statistical significance levels compared to IL-8 production by cells incubated with drug-untreated mixed biofilm supernatant (L0) were marked ** for *p* < 0.01, and *** for *p* < 0.001

**Fig. 6:**
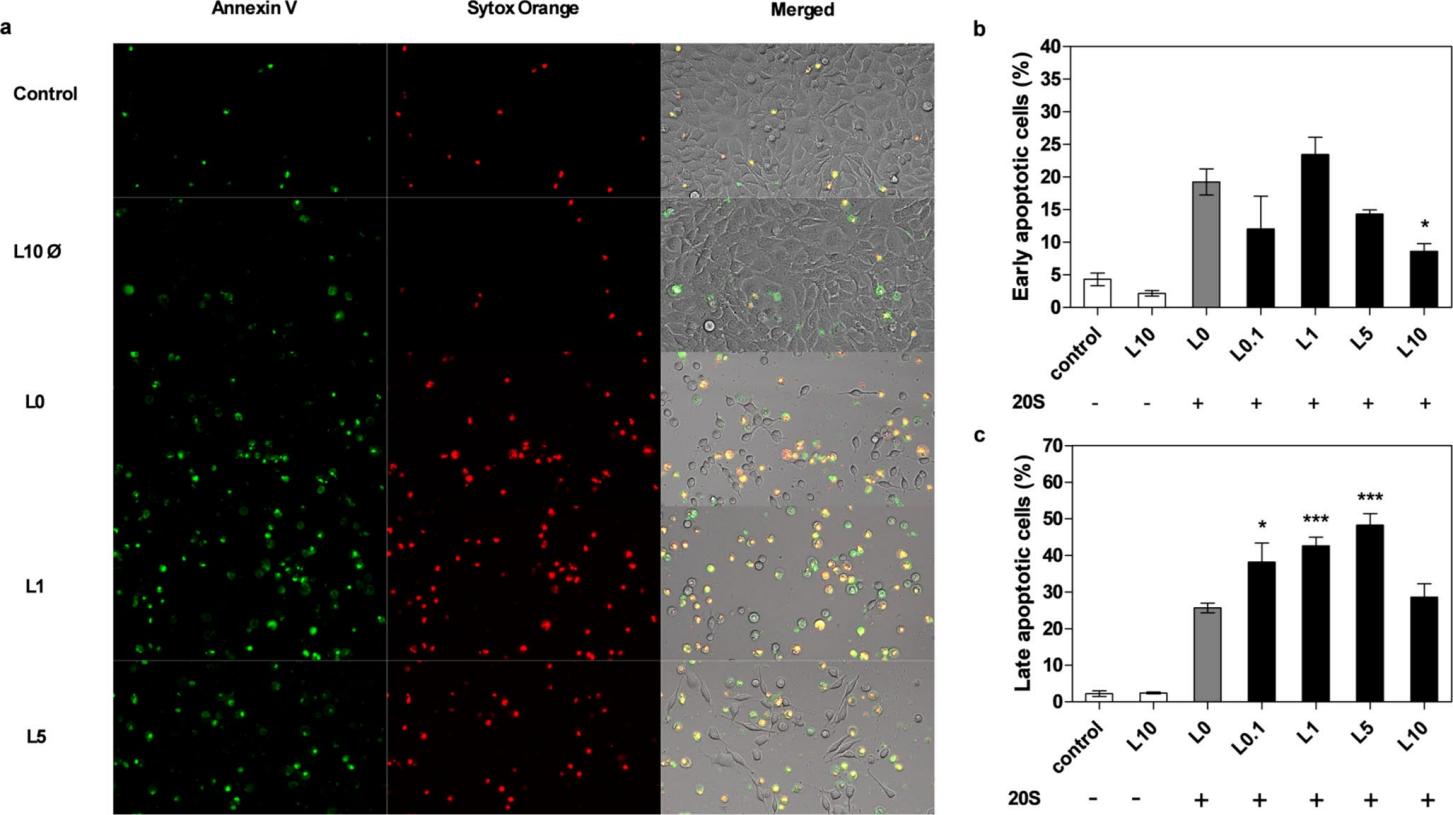
Effect of supernatant from mixed biofilm treated with levofloxacin on early and late apoptosis of BEAS-2B human epithelial cells. BEAS-2B cells were incubated for 24 h in RPMI 1640 with 20% supernatants (20 S) collected from mixed biofilms treated with levofloxacin; control – RPMI 1640 with 2% FBS; L10Ø – levofloxacin (10 µg/ml) in RPMI 1640 with 2% FBS; L0 – supernatant from biofilm non-treated with levofloxacin; L.01, L1, L5, L10 – supernatant from biofilm treated with levofloxacin at a concentration of 0.1, 1, 5, 10 µg/ml. Early apoptotic cells were stained with Annexin V (green), and late apoptotic cells were stained with Sytox Orange (red). Representative images from microscopic analysis in the FITC, mCherry, and brightfield channels (**a**) are presented. Early (**b**) and late (**c**) apoptotic cells were counted using ImageJ software. The data are presented as mean ± SEM. For the statistical analysis one-way ANOVA test with Dunnett’s multiple comparisons was used. The results were considered statistically significant when compared to L0 and marked with * for *p* < 0.05, and *** for *p* < 0.001

**Fig. 7:**
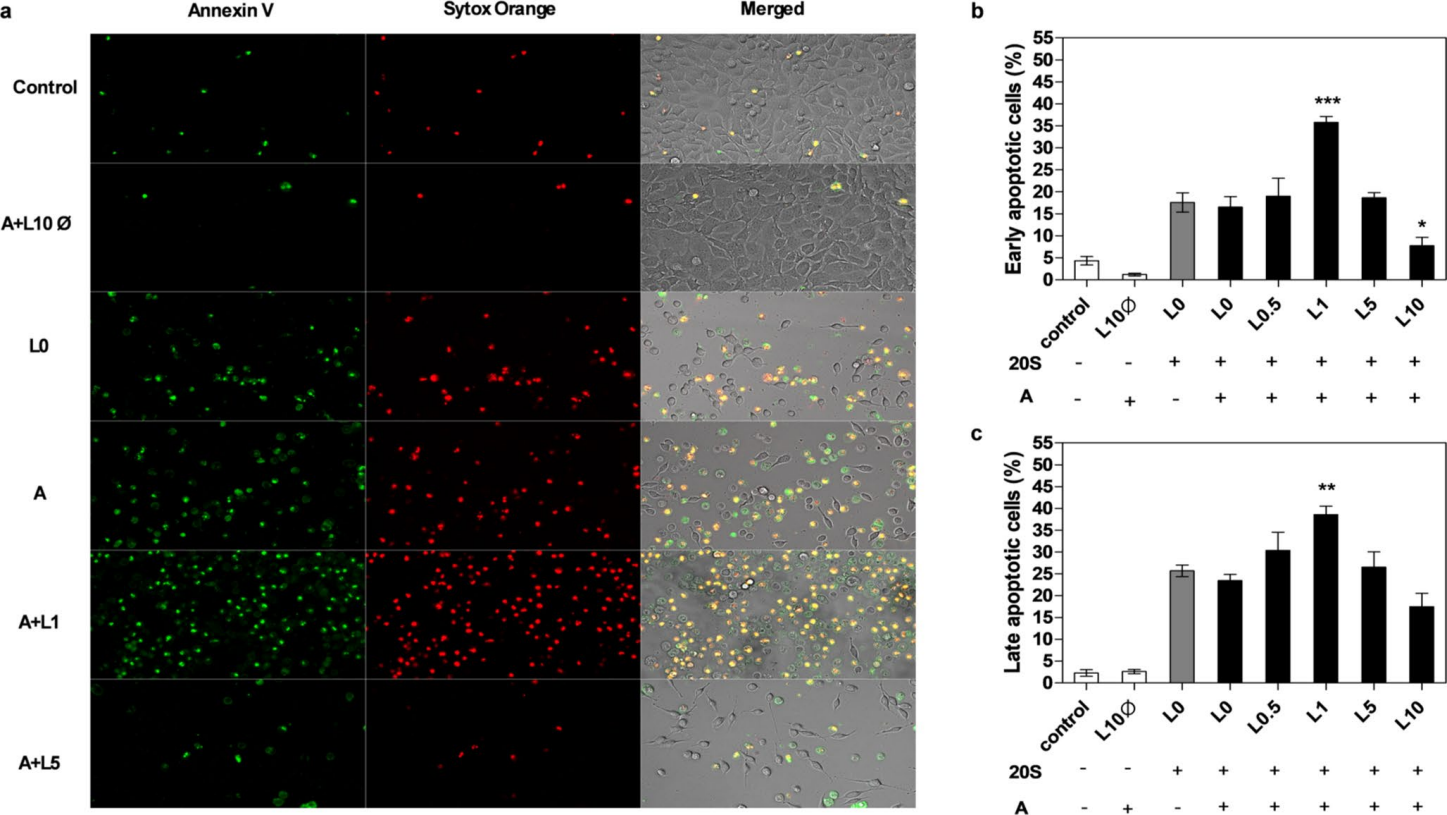
Influence of supernatants collected from mixed biofilms treated with levofloxacin and amphotericin B on early and late apoptosis of BEAS-2B human epithelial cells. BEAS-2B cells were cultured for 24 h in RPMI 1640 supplemented with 20% supernatants (20 S) collected from dual-species biofilms treated with levofloxacin (L0.5, L1, L5, L10 corresponding to a concentration of 0.5, 1, 5, 10 µg/ml) and amphotericin B (A – 0.5 µg/ml); control – RPMI 1640 with 2% FBS; L10Ø – levofloxacin (10 µg/ml) in RPMI 1640 with 2% FBS; L0 – supernatant from biofilm non-treated with levofloxacin. Early and late apoptotic cells were stained (**a**) with Annexin V (green), and Sytox Orange (red), respectively, and counted (**b**, **c**) using ImageJ software. The data shown are presented as mean ± SEM. For the statistical analysis one-way ANOVA test with Dunnett’s multiple comparisons was used. The results were considered statistically significant when compared to effect of supernatant from biofilm non-treated with levofloxacin and amphotericin B and marked with * for *p* < 0.05, ** for *p* < 0.01, and *** for *p* < 0.001

To verify the consequences for host cells of a biofilm subjected to antibiotic treatment, we selected an example using levofloxacin. Despite the death of bacterial cells treated with this antibiotic, traces of their presence in the environment were identified by increased gingipain activity released into the medium. In our model of lung epithelial cells (BEAS-2B), we observed a significant increase in interleukin-8 (IL-8) production, that far exceeds the response of cells in contact with an untreated biofilm (Fig. 5A). Moreover, this effect was proportional to the presence of gingipains in the environment rather than the number of infecting bacterial cells. These observations also correlated with a twofold increase in early apoptosis in the cells, as determined by Annexin V staining, and their progression into the late apoptosis pathway, as determined by Sytox Orange staining for levofloxacin concentrations correlating with the highest level of gingipain release in the biofilm environment (Fig. 6). Similar considerations were made for the use of mixed therapies involving levofloxacin and amphotericin B, which more effectively eliminate yeast cells in the mixed biofilm but does not significantly impact the potential of released gingipains. The use of combined antibiotic and antifungal therapy resulted in a several-fold increase in IL-8 production upon contact with epithelial cells (Fig. 5B) and a twofold increase in the number of late apoptotic cells (Fig. 7).

## Discussion

The vast majority of microbial infections are associated with the formation of highly structured and dynamically developing multispecies biofilms encased in an extracellular matrix, comprising a complex combination of different microbe types [35]. The multidirectional interaction between microbial communities can not only influence the behaviour of the microbiome but also govern biofilm-based resistance to conventional pharmaceutical treatments [36].

Using an approach of mixed in vitro biofilm system created in the AP model and a set of drugs commonly used in AP therapy, we considered aspiration that might contain both yeast and bacteria, which can form mixed-species biofilms in the upper respiratory tract. In particular, we highlighted the possibility of the presence of *P. gingivalis* bacteria, which are prevalent in oral cavity of individuals with periodontitis, as a special case. This selection was based on the suggested correlation between the presence of this bacterium and other systemic diseases [37]. *C. albicans* was long overlooked as a bystander in polymicrobial biofilm-associated infections. However, reports from recent years indicate that *C. albicans* plays a pivotal role in mixed-species biofilms by supporting the growth and enhancing the virulence of co-existing bacteria through metabolic cross-feeding, physical attachment, and modulation of the biofilm environment [38]. It interacts with oral bacteria such as *Streptococcus gordonii* [39], *Fusobacterium nucleatum* [40], or *Pseudomonas aeruginosa*, influencing biofilm formation and possibly increasing resistance to antimicrobial treatments [41]. *C. albicans* also employs various virulence factors, including surface adhesins, to facilitate coexistence with *P. gingivalis*, enhancing biofilm stability and pathogenicity [42], as well as modulating host immune responses [29]. These interactions may complicate the clinical management of infections, underscoring the crucial need for understanding these processes to develop effective therapeutic strategies to manage polymicrobial infections.

The appropriate selection of antimicrobials for the treatment of AP is challenging and depends on its aetiology, but the most often applied treatment employs antibiotics with broad activity towards anaerobes [43–45]. The antibiotics we selected for the in vitro biofilm model approach include metronidazole, levofloxacin, meropenem and vancomycin. Metronidazole works by disrupting bacterial DNA, while levofloxacin - inhibiting critical enzymes involved in its processing: DNA gyrase and topoisomerase IV [46, 47]. Meropenem and vancomycin act on bacterial cell wall integrity, where meropenem can be covalently bound to penicillin-binding proteins involved in the biosynthesis of mucopeptides responsible for the stabilization of the bacterial cell wall [48]. Bactericidal properties of vancomycin result from the prevention of proper formation of the peptidoglycan matrix and cell wall biosynthesis, which alters cell membrane permeability. It also influences bacterial RNA synthesis [49]. Among them, metronidazole is considered the gold standard for treating anaerobic infections, with particular effectiveness against *P. gingivalis* in treating periodontal infections [50]. While not conventionally used for anaerobic infections, levofloxacin has shown efficacy against various anaerobic pathogens. However, it is less commonly studied specifically for *P. gingivalis* compared to metronidazole, but its combined usage with other antibiotics has been suggested to improve treatment outcomes for mixed bacterial infections [51]. On the other hand, *P. gingivalis* can develop resistance to levofloxacin during adaptation [52]. Meropenem is highly effective against a wide range of Gram-negative and Gram-positive bacteria, including anaerobes, and is useful in the treatment of hospital-acquired pneumonia, demonstrating good in vitro activity against *P. aeruginosa* and some enterococci [53]. Vancomycin is particularly effective against Gram-positive bacteria, including MRSA and other staphylococci, and is less effective against Gram-negative bacteria and anaerobes like *P. gingivalis* [54] and serves as an example of non-specific therapy in this case.

Considering the presented results, it appears that prior to bacterial cell death induced by antibiotic treatment, *P. gingivalis* cells in mixed biofilms produced gingipains, which were more efficiently released into the medium compared to untreated bacterial cells. It seems, however, that in the case of meropenem treatment, the influence of this antibiotic on the release of gingipains was much slower and occurred over a wider range of concentrations. This indicates that the bacterial proteolytic potential released during the formation of the mixed biofilm was maintained much longer in such a mixed culture, as indicated by the diminishing presence of bacteria, which may pose a threat to the host cells surrounding the site of infection. Moreover, this slow release of gingipains was likely the reason for its negligible effect on fungal cell viability and the metabolism of mixed-species biofilms.

Among the antifungal drugs tested herein in relation to mixed biofilms, were fluconazole, caspofungin and amphotericin B. The mechanism of action of fluconazole involves the interference with ergosterol synthesis by binding to cytochrome P-450, which consequently leads to the disruption of the integrity of the fungal cell membrane. Furthermore, fluconazole can also contribute to chitin synthesis damage, leading to the destabilization of the fungal cell wall [55]. Another antifungal drug, caspofungin, a representative of echinocandins, inhibits the synthesis of beta-(1,3)-D-glucans, an essential component of *C. albicans* cell wall, by blocking beta-(1,3)-glucan synthase [56]. The last studied drug was amphotericin B, which has fungistatic or fungicidal properties, depending on the concentration and fungal susceptibility. These properties result from its irreversible binding to ergosterol, leading to the formation of transmembrane channels, increased membrane permeability, leakage of intracellular components, and ultimately fungal cell death. Amphotericin B can also induce the production of reactive oxygen species (ROS) within fungal cells, leading to oxidative stress contributing to cell death by damaging cellular components [57]. Based on the results presented herein, the metabolic activity of the mixed biofilm after the addition of fluconazole or caspofungin was significantly increased compared to the single-species fungal biofilm, probably due to the good condition of the bacterial cells living in the formed biofilm, indicating a well-growing community that protects bacterial cells. Under amphotericin B treatment, the metabolic activity in the mixed biofilm was reduced rather similarly to the fungal monoculture, without alterations in bacterial cell viability or gingipain activity. This suggests that the enhanced effectiveness of amphotericin B may be due to structural changes in the cell wall and membrane caused by the destructive actions of proliferating bacteria. It seems that the condition of yeasts in the treated mixed biofilm is no longer as crucial for the proliferating bacteria, which, due to the initial environmental modifications by the yeast, can gain a new niche for colonization. However, analyzing the potential for further bacterial proliferation under these conditions would require long-term observation.

In the model we applied, as anticipated, mixed biofilms involving both studied microorganisms demonstrated increased resistance to the action of both antibiotics and antifungals. However, we would like to highlight the important and novel aspects of our research. The first concerns the potential for maintaining the enhanced killing capability of bacteria within the mixed biofilm, represented by the secreted proteases of *P. gingivalis*, even during partial or complete bacterial death resulting from antibiotic therapy. The mechanisms of gingipain release by *P. gingivalis* into the environment primarily involve the type IX Secretion System, which recognizes and transports gingipains from the cytoplasm to the cell surface and eventually into the extracellular environment [58]. Although some gingipains are anchored to the cell surface through lipid modifications or protein-protein interactions, these membrane-bound gingipains can directly interact with host tissues and immune cells, facilitating localized tissue destruction and immune modulation [59]. These enzymes are also packaged into extracellular membrane vesicles (EVs), which carry gingipains and other virulence factors, protecting them from degradation and enabling their delivery to the microenvironment [60]. This method of EVs delivery enhances the efficiency of gingipain action on host tissues and cells [61]. Moreover, EVs contribute to biofilm formation by providing structural components and promoting bacterial community development. Although it is difficult to estimate which pool of the enzyme activity in the supernatant is detected by our method using a small molecule substrate, and the mixed biofilm, even after multiple washes, still released gingipains and exhibited partial activity of these enzymes (Fig. 2 in Supplementary data). Considering the fact that increased release of gingipains into the biofilm environment was observed under the influence of less specific drugs that affect bacterial cell membrane stability, it can be assumed that antibiotics, as a type of stress factor, may influence the increased production and release of EVs by *P. gingivalis*. This possibility was noticed, for example, for methicillin-resistant *Staphylococcus aureus* (MRSA) under exposure to sub-inhibitory concentrations of ampicillin [62], and for *Escherichia coli* under antibiotic stress [63]. Moreover, gingipains can not only be released into the environment, but can also be captured by the formed mixed-species biofilm due to the possible interactions of gingipain RgpA with the surface proteins of *C. albicans*—such as the member of the agglutinin-like sequence (Als) family, Als3, or Mp65—adhesins commonly occurring in biofilms, and enolase, which has the new function of a moonlighting protein on the fungal hyphae surface, as we documented in our previous work [20]. Given the presence of gingipain R both on the bacterial cell surface and in the secretome, as well as its ability to interact with *C. albicans* proteins, the examination of the activity of this particular proteinase in treated mixed biofilms was selected to determine the level of activity of bacterial cells in this study. It is worth noting that Als3 also interacts with another *P. gingivalis* protein, InlJ, which facilitates the coadhesion of these organisms and the upregulation of *P. gingivalis* genes associated with growth, division, and virulence [21].

Our research demonstrated that ineffective antibiotic therapy significantly increased gingipain activity within the biofilm. This increase exacerbated inflammatory symptoms, as indicated by elevated IL-8 levels, and led to the death of epithelial cells. Such changes were also documented in the work of Farrugia et al. [60], which showed that gingipains degrade crucial structural proteins, such as E-cadherin, cytokeratins, and junctional adhesion molecules [64], weakening the integrity of the epithelial barrier and facilitating microbial invasion and colonization [65]. Moreover, gingipains can induce the expression and activation of matrix metalloproteinases (MMPs) in gingival epithelial cells, which degrade extracellular matrix components, leading to further tissue destruction and loss of structural integrity in the periodontium [66]. Gingipains also degrade key immune signalling molecules, such as cytokines and their receptors, impairing the host’s ability to mount an effective immune response. This immune modulation allows *P. gingivalis* to persist in the periodontal pocket and exacerbate tissue damage [67–69]. These enzymes also induce the production of reactive oxygen species (ROS) within gingival epithelial cells, leading to oxidative damage and apoptosis, and further contributing to tissue destruction [70]. They also induce epigenetic changes such as DNA methylation and histone modifications, which alter gene expression patterns in gingival epithelial cells, leading to long-term alterations in cellular behaviour, contributing to chronic inflammation and disease progression. Importantly, from the perspective of our research, such disruption of the epithelial barrier and alteration of the local environment by gingipains might create conditions that favour the colonization and invasion of other pathogenic microorganisms, leading to co-infections that can complicate the clinical management of disease, as was also shown for collaboration of *P. gingivalis* with *Fusobacterium nucleatum* [71]. Therefore, a comprehensive understanding of the mechanism and effectiveness of antimicrobial therapy underscores the importance of targeting gingipains in therapeutic strategies for non-periodontal diseases in which *P. gingivalis* can collaborate with other microbes.

The issue of the increased effectiveness of antifungal treatment with amphotericin B in mixed-species biofilm compared to single-species fungal biofilm, which we observed, requires separate consideration. Several factors may contribute to this phenomenon. Bacterial compounds secreted into the environment, such as enzymes or metabolites, can modify the biofilm matrix, affecting the penetration and activity of amphotericin B, and leading to gradual fungal cell death. Another explanation involves the possible interaction of bacterial secreted compounds with the fungal cell surface or their influence on fungal cell metabolism. For example, *P. aeruginosa* increases the susceptibility of *C. albicans* to amphotericin B by inducing oxidative stress through secreted phenazines, which weakens the fungal cells and makes them more susceptible to the antifungal agent [72]. Moreover, these bacteria downregulate key detoxification enzymes of *C. albicans*, preventing an effective response to oxidative stress induced by amphotericin B, thereby increasing fungal cell death.

To explain our observation, we can consider that proteolytic enzymes produced intensively by protected bacterial cells might be responsible for this effect. Indeed, comparing the influence of amphotericin B on *C. albicans* viability in biofilms formed with *P. gingivalis* wild-type W83 strain and its gingipain-deficient mutant (*∆K∆RAB*) showed that the effectiveness of antifungal agent increases only when bacteria in the biofilm can release gingipains. The effect of amphotericin B against a monomicrobial yeast biofilm and a mixed biofilm involving the bacterial mutant strain was similar (Fig 3  in Supplementary data). However, it is difficult to determine whether this is due to the degradation of proteins involved in biofilm matrix formation or the degradation of surface proteins of *C. albicans* cells, mainly mannoproteins responsible for the stabilization of the fungal cell wall. On the other hand, our preliminary analysis of the genes encoding enzymes involved in fungal cell detoxification (Cat1, Sod5), which expression increased in mixed species biofilms, suggest that these processes are activated in contact with *P. gingivalis*, only in part by gingipains (Fig. 4  in Supplementary data). In any case of amphotericin B usage, the consequence of contact with bacteria possessing such high proteolytic potential is the gradual death of yeast cells, especially their filamentous forms, as determined from our microscopic observation. It is worth noting that these fungal cell forms produce adhesins important for interactions of both microorganisms, and their destruction may also be important for further bacterial dissemination to new niches. However, elucidating the detailed mechanisms of such complex interactions requires further research.

The findings presented pertain to an in vitro model of a biofilm formed by two microorganisms. However, it is important to note that in vivo conditions may additionally alter these interactions due to various local or systemic factors, or the presence of other microorganisms. The diversity of microorganisms at the site of infection necessitates considering the actions of larger microbial consortia in therapy planning, as biofilm formation enhances their stability and resistance to antibiotics. Special attention should be given to yeasts, which can transform from typical commensals to pathogens under conditions of weakened immunity. The biofilm they form, with its highly developed matrix, provides strong protection not only for the yeasts, but also for coexisting bacteria. This protective action can render commonly used pharmaceuticals ineffective. Additionally, the deregulation of the killing potentials of pathogens can unexpectedly exacerbate ongoing infections. Understanding the mutual interactions of pathogens during infection will enable better-targeted therapies and potentially identify new therapeutic targets for mixed infections. Additionally, it is also important to emphasize that the sequence and dosage of antimicrobial compounds can significantly impact their effectiveness.

## Electronic supplementary material

Below is the link to the electronic supplementary material.

Supplementary Material 1

## Data Availability

No datasets were generated or analysed during the current study.
