# Supplementary material for: The efficacy of antimicrobial therapies in the treatment of mixed biofilms formed between Candida albicans and Porphyromonas gingivalis during epithelial cell infection in the aspiration pneumonia model
Source: Med Microbiol Immunol. 2025 Feb 4;214(1):8. doi: 10.1007/s00430-025-00818-2 (PMC11794384; doi:10.1007/s00430-025-00818-2)
Supplement: Supplementary file 1 — Supplementary Material 1 [file 430_2025_818_MOESM1_ESM.docx]

**The efficacy of antimicrobial therapies in the treatment of mixed biofilms formed between *Candida albicans* and *Porphyromonas gingivalis* during epithelial cell infection in the aspiration pneumonia model**

**Journal name: Medical Microbiology and Immunology**

Grazyna Bras^1‡^, Ewelina Wronowska^1‡^, Miriam Gonzalez-Gonzalez^1,2^, Magdalena Juszczak^1,2^, Magdalena Surowiec^1,2^, Wiktoria Sidlo^1^, Dorota Satala^1^, Kamila Kulig^1^, Justyna Karkowska-Kuleta^1^, Joanna Budziaszek^3^, Joanna Koziel^3^, Maria Rapala-Kozik^1*^

^1^ Department of Comparative Biochemistry and Bioanalytics, Faculty of Biochemistry, Biophysics and Biotechnology, Jagiellonian University, Gronostajowa 7, 30-387 Kraków, Poland

^2^ Doctoral School of Exact and Natural Sciences, Faculty of Biochemistry, Biophysics and Biotechnology, Jagiellonian University, Gronostajowa 7, 30-387 Kraków, Poland

^3^ Department of Microbiology, Faculty of Biochemistry, Biophysics and Biotechnology, Jagiellonian University, Gronostajowa 7, 30-387 Kraków, Poland

^*^Correspondence: maria.rapala-kozik@uj.edu.pl

**Supplementary Data**

**1. Verification of the effectiveness of selected antibiotics in the studied system against *Porphyromonas gingivalis* bacteria proliferating under anaerobic conditions**

The effectiveness of antibiotics against *P. gingivalis* W83 strain was assessed by culturing the bacteria in RPMI 1640 medium under anaerobic conditions. These assessments were essential to compare variability within mono- and dual-species biofilms formed in the presence of 10% FBS.

Based on the data presented in Fig.1, the optimal bactericidal concentrations of individual antibiotics on the bacterial monoculture were determined. The results are presented as the minimum inhibitory concentration leading to 90% of culture death (MIC90) and compared with literature values presented for culture conditions not including FBS (Table 1).





**Fig. 1 The influence of antibiotic treatment on *P. gingivalis* biofilm formed under anaerobic condition in medium containing 10% FBS**

*P. gingivalis* cells (10⁸ cells/ml) were plated in RPMI 1640 with 10% FBS in the wells of a 96-well microplate at 37°C for 24 hours in a GENbox jar anaerobic generator (bioMérieux, Craponne, France). Then antibiotic was added at specified concentration in the range of 0-50 µg/ml and the bacterial culture was incubated for the next 24 hours under the same conditions as before treatment.To determine the survival rate of bacterial cells in biofilms, CFUs were analyzed after washing the biofilm once with PBS. In the plot, 100% survival rate of *P. gingival*is is represented by bacteria not treated with antibiotics.

**Table 1 Comparison of MIC90 values for the activity of selected antibiotics against monocultures of *P. gingivalis* grown in the presence of 10% FBS under anaerobic conditions**

| **antibiotic** | **determined MIC90 (μg/ml)** | **literature MIC value (μg/ml)*** |
| --- | --- | --- |
| meropenem | 1 | 1 [1] |
| metronidazole | 5 | 3.12 [2] |
| levofloxacin | 0.1 | 0.2 [3] |
| vancomycin | 1 | 4 [4] |

**2. Comparison of gingipain activity in dual-species culture formed by *P. gingivalis*** **and *C. albicans*** **under aerobic conditions**

Since we used gingipain R activity as an indicator of bacterial viability, we compared the presence of this activity in the supernatant of the culture medium, in the floating cells of *P. gingivalis,* and in the biofilm washed three times with PBS. The cultures were conducted under aerobic conditions, as described in the Materials and Methods section. The results are presented in Fig 2.


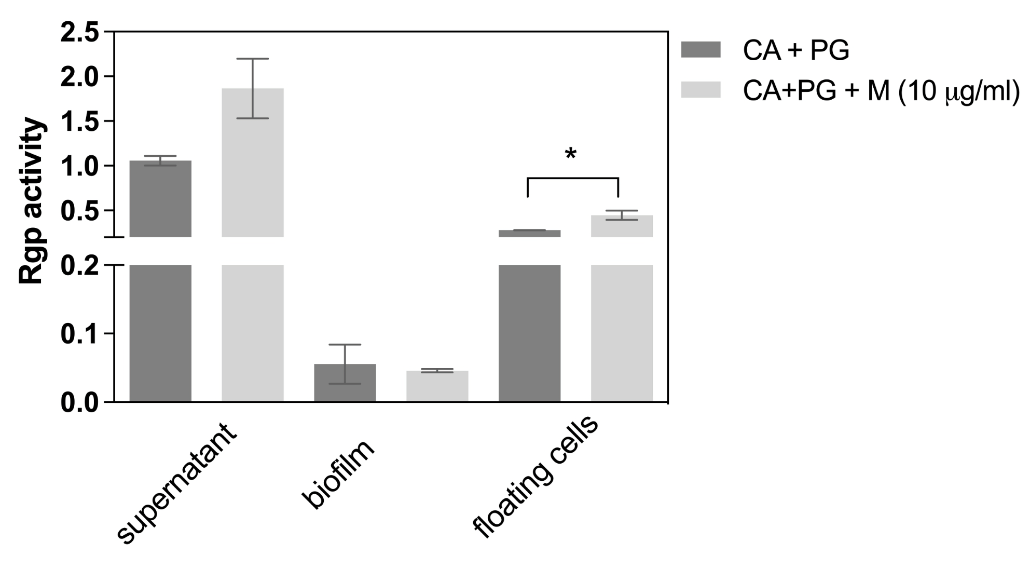


**Fig. 2 Impact of meropenem treatment on gingipain activity in biofilm structure, floating bacterial cells of dual-species *P. gingivalis* and *C. albicans***

C. albicans (10^7^ cells/ml) and P. gingivalis (10^8^ cells/ml), suspended in RPMI 1640 medium supplemented with 10% FBS, were plated into the wells of a 96-well microplate and cultured for 24 hours at 37°C under aerobic conditions to form single- or mixed-species biofilms. After this time, the biofilms were treated with meropenem (M) at the final concentration of 10 µg/ml, and then incubated for the next 24 hours. The gingipain R (Rgp) activity was measured in supernatants collected from above the biofilms, in floating bacterial cells collected, washed and suspended in PBS, and in the washed biofilm, using the substrate BApNA. Representative results from five independent experiments are presented as means ± standard error. Statistical analysis was performed using a one-way ANOVA with Dunnett’s post hoc test to compare the means of groups (* p < 0.05)

The main pool of gingipains is released into the supernatant, as shown in the figure. A significant portion of cell-associated gingipains, retaining their activity, is found in the floating cells, while the smallest, yet environmentally significant portion, is associated with the biofilm. This suggests that most of the bacterial cells proliferating in contact with *C. albicans*, and carrying their virulent potential (gingipains), remain mobile in the presence of biofilm, ready to colonize new, more favorable niches. However, the biofilm itself retains gingipain activity, either through cells associated with fungal filaments or as an enzyme bound to yeast adhesins. Regardless of the biofilm fraction considered (cellular or supernatant), the presence of the antibiotic at the selected concentration leads to increased production of gingipains.

**3. Gingipains enhance the effectiveness of amphotericin B in reducing the viability of yeast cells**

The susceptibility of *C. albicans* cells to amphotericin B treatment was examined in both mono- and dual-species biofilms formed with the *P. gingivalis* W83 wild-type strain and its isogenic gingipain-null mutant, ∆K∆RAB (lacking gingipains Kgp, RgpA, and RgpB, as described in the Materials and Methods section). The data presented in the Fig 3 show that the effectiveness of amphotericin B against a monomicrobial yeast biofilm and a mixed biofilm involving the mutant is similar, and the effectiveness of the antifungal agent increases only when the bacteria within the biofilm are capable of releasing gingipains.


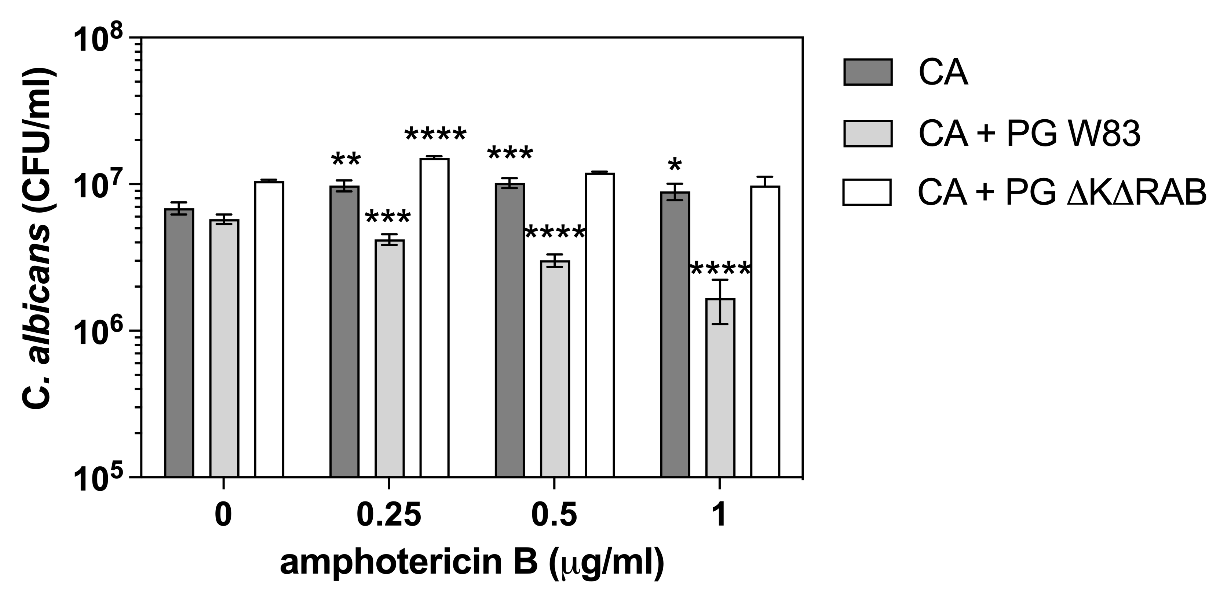


**Fig. 3 *C. albicans* cell viability within the mono- and dual-species biofilm formed with *P. gingivalis***

C. albicans cells (10^7^ cells/ml) and P. gingivalis (10^8^ cells/ml) wild type and an isogenic gingipain-null mutant strain, ∆K∆RAB, were suspended in RPMI 1640 medium supplemented with 10% FBS, placed to the wells of a 96-well microplate and cultured for 24 hours at 37°C under aerobic conditions to form single- or mixed-species biofilms. Then, the biofilms were treated with amphotericin B at the final concentration range 0-1 µg/ml, and incubated for the next 24 hours. The biofilms were washed, scraped, and after dilution, plated on YPD agar plates to determine CFU/ml of C. albicans. Representative results from five independent experiments are presented as means ± standard error. Statistical analysis was performed using a one-way ANOVA with Dunnett’s post hoc test to compare the means of groups (* p < 0.05; ** p < 0.01; *** p < 0.001; **** p < 0.0001)

**4. Gene expression of fungal cell protecting proteins in mixed-species biofilm.**

For analysis of expression of selected genes (*CAT1,* *SOD5, HSP90* and *ACT1* as housekeeping gene), fungal RNA was isolated from biofilm forming cells after 24 hours of propagation at 37°C in a flow model, described by Zarnowski et al., [5]. Mono-species biofilms of *C. albicans* (10^6^ cells/ml), and dual-species biofilm of *C. albicans* (10^6^ cells/ml) and P*. gingivalis* W83 wild-type strain (10^7^ cells/ml) or its isogenic gingipain-null mutant, ∆K∆RAB (10^7^ cells/ml) were formed in RPMI 1640 medium with or without FBS. The cells were scraped from the bottles, suspended in PBS, and centrifuged. The cell pellet was collected. All samples were prepared in three technical replicates.

Total RNA was isolated from cells using Tri Reagent (Merck Millipore, Burlington, MA, USA) following standard protocols, with initial cell homogenization performed in the Precellys® Evolution tissue homogenizer (Bertin Technologies SAS, Montigny-le-Bretonneux, France). cDNA synthesis was carried out using the M-MLV Reverse Transcriptase kit (Promega, Madison, WI, USA) according to the manufacturer's instructions. Gene expression analysis was conducted using the QuantStudio™ 3 Real-Time PCR System (Thermo Fisher Scientific). Amplification of cDNA occurred at an annealing temperature of 58°C with Kappa SYBR Green Master Mix (Merck Millipore) and specific primers (Table 2). Relative gene expression was calculated using the 2-ΔΔCt method [6], normalized to the *ACT1*  reference gene and the values obtained for control samples (*C. albicans* cells not treated with additional factors).

**Table 2 The list of primers used for quantification of genes encoding: *ACT1* – actin;  *CAT1* – catalase; *SOD5* – superoxide dismutase 5; *HSP90* – heat shock protein 90**

| **Gene** | **Primer forward 5’→ 3’** | **Primer reverse 5’ → 3’** | **Reference** |
| --- | --- | --- | --- |
| *ACT1* | GATTTTGTCTGAACGTGGTTAACAG | GGAGTTGAAAGTGGTTTGGTCAATAC | [7] |
| *CAT1* | GGAGTTGAAAGTGGTTTGGTCAATAC | GTGAGTTTCTGGGTTTCTCTT | [7] |
| *SOD5* | ATCTTACATTGGCGGTTTAT | GACCATTTACTACTGCTCTCTCA | [8] |
| *HSP90* | GCTGACGTTTCTATGATTG | CATCCAAAGTAACAGTGAAC | [9] |

The preliminary analysis of the level of the expression of genes encoding enzymes involved in fungal cell detoxification (*CAT1, SOD5, HSP90*) showed increased expression of these genes in the presence of FBS, and additionally in contact with both *P. gingivalis* strains, although with less efficiency for the wild type W83 strain compared to its mutant deficient in gingipain production. The genetic results are supported by mass spectrometry analysis of proteins presented in biofilm matrix (unpublished data).


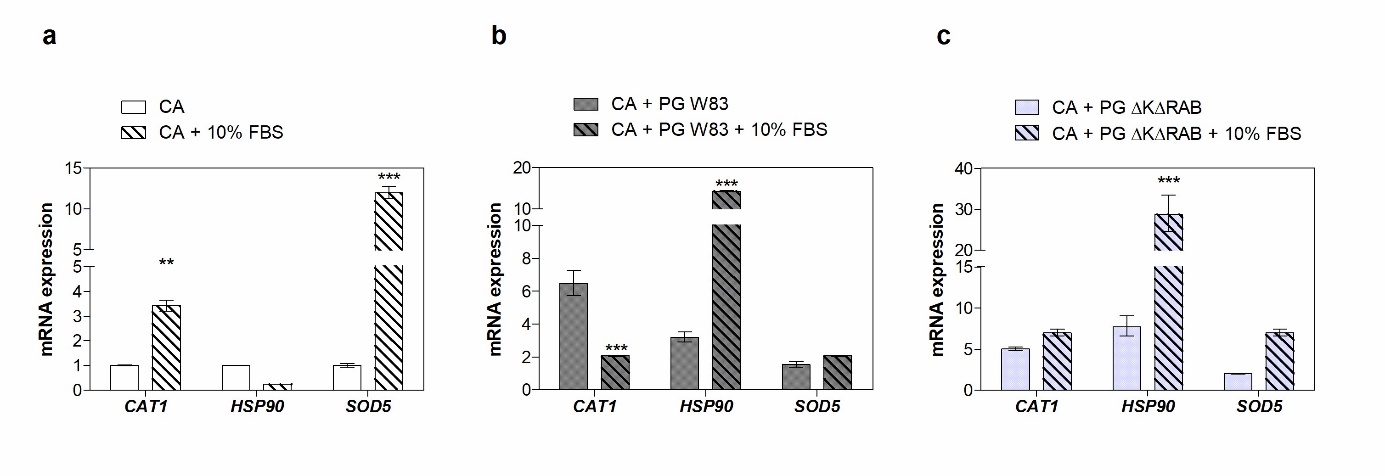


**Fig. 4 Expression levels of selected fungal genes important in stress response**

Left panel shows the impact of 10% FBS on mRNA levels of selected genes, while middle and right panels illustrate the influence of bacteria on the expression of these genes. The graphs related to bacterial influence present results normalized to homotypic biofilms, without and with 10% FBS, for which the expression value was set to 1. The graphs show representative results obtained from five biological replicates for cultures without FBS and three for cultures with added FBS. Statistical analysis was performed using ANOVA with Bonferroni's test, with significance levels indicated as **p<0.01, ***p<0.001 (comparison to *C. albicans* biofilm).

**REFERENCES**

1. Santos FA, Bastos EMA, Rodrigues PH, de Uzeda M, de Carvalho MAR, de Macedo Farias L, Moreira ESA (2002) Susceptibility of *Prevotella intermedia/Prevotella nigrescens* (and *Porphyromonas gingivalis*) to Propolis (Bee Glue) and other Antimicrobial Agents. Anaerobe, 8: 9–15. doi: 10.1006/anae.2002.0411.

2. Fournier-Larente J, Morin MP, Grenier D (2016) Green tea catechins potentiate the effect of antibiotics and modulate adherence and gene expression in *Porphyromonas gingivalis*. Arch Oral Biol, 65: 35–43. doi: 10.1016/j.archoralbio.2016.01.014.

3. Eguchi T, Shimizu Y, Furuhata K, Fukuyama M (2002) Antibacterial Activity of New-quinolone and Macrolide Antibiotics against Oral Bacteria. J Jpn Assoc Infect Dis, 76(11): 939–945. doi: 10.11150/kansenshogakuzasshi1970.76.939.

4. Citron DM, Tyrrell KL, Merriam CV, Goldstein EJ (20120 In vitro activities of CB-183,315, vancomycin, and metronidazole against 556 strains of *Clostridium difficile*, 445 other intestinal anaerobes, and 56 *Enterobacteriaceae species*. Antimicrob Agents Chemother, 56(3):1613-5. doi: 10.1128/AAC.05655-11.

5. Zarnowski R, Sanchez H, Covelli, AS, Dominguez E, Jaromin A, Bernhardt J, Mitchell KF, Heiss C, Azadi P, Mitchel, A, Andes DR (2018). *Candida albicans* biofilm-induced vesicles confer drug resistance through matrix biogenesis. PLoS biol, 16: e2006872. https://doi.org/10.1371/journal.pbio.2006872.

6. Livak KJ, Schmittgen TD (2001). Analysis of relative gene expression data using real-time quantitative PCR and the 2(-Delta Delta C(T)) Method. Methods (San Diego, Calif.), 25: 402–408. https://doi.org/10.1006/meth.2001.1262

7. Wolak N, Tomasi M, Kozik A, Rapala-Kozik M (2015). Characterization of thiamine uptake and utilization in *Candida* spp. subjected to oxidative stress. Acta Biochim. Pol*.* 62: 445–455. https://doi: 10.18388/abp.2015_1044,

8. Guevara-Lora I, Bras G, Juszczak M, Karkowska-Kuleta J, Gorecki A, Manrique-Moreno M, Dymek J, Pyza E, Kozik A, Rapala-Kozik M (2023). Cecropin D-derived synthetic peptides in the fight against *Candida albicans* cell filamentation and biofilm formation. Front Microbiol. 13:1045984. https://doi: 10.3389/fmicb.2022.1045984.

9. Garnaud C, García-Oliver E, Wang Y, Maubon D, Bailly S, Despinasse Q, Champleboux M, Govin J, Cornet M (2018). The Rim Pathway Mediates Antifungal Tolerance in *Candida albicans* through Newly Identified Rim101 Transcriptional Targets, Including Hsp90 and Ipt1. Antimicrob Agents Chemother 62: e01785-17. https://doi.org/10.1128/AAC.01785-17
